# Development of intelligent tools to predict neuroblastoma risk stratification and overall prognosis based on multiphase enhanced CT and clinical features

**DOI:** 10.3389/fneur.2025.1573398

**Published:** 2025-06-19

**Authors:** Wei Zhao, Yahui Han, Xiaokun Yu, Jianing Liu, Jiao Zhang, Juan Li

**Affiliations:** ^1^Department of Pediatric Surgery, The First Affiliated Hospital of Zhengzhou University, Zhengzhou, China; ^2^Department of Respiratory Medicine, The First Affiliated Hospital of Zhengzhou University, Zhengzhou, China

**Keywords:** deep learning, swin transformer, neuroblastoma, multiphase enhanced CT, prognostic

## Abstract

**Background:**

Neuroblastoma (NB) is a common malignancy in children, and accurate risk stratification and prognostic assessment are essential for personalized treatment. Current tumor assessment methods rely on clinical features and conventional imaging techniques, which have limited predictive accuracy. The aim of this study was to develop a deep learning model based on multiphase enhanced CT images and clinical features to improve the accuracy of risk stratification and prognostic assessment of NB.

**Methods:**

Multi-phase enhanced CT images and clinical features from 202 NB patients were collected. Four risk stratification classifiers were developed using the Swin Transformer model and evaluated in training and testing cohorts. Prognostic models were constructed using a combination of multiple machine learning algorithms in conjunction with CT image features and clinical characteristics.

**Results:**

Swin-ART based on arterial phase images was the best risk stratification classifier with an AUC of 0.770 (95% CI: 0.613–0.909) and an accuracy of 0.780 in the testing cohort. In the prognostic assessment, the combined model of backward stepwise Cox regression and randomized survival forest (RSF) obtained the highest mean C-index of 0.84. The 1-, 3-, and 5-year AUC values of the optimal prognostic model in the training cohort were 0.93 (95% CI: 0.927–0.942), 0.93 (95% CI: 0.929–0.946), and 0.96 (95% CI: 0.953–0.974), respectively. The corresponding AUC values for the testing cohort were 0.90 (95% CI: 0.857–0.934), 0.87 (95% CI: 0.808–0.928), and 0.91 (95% CI: 0.718–0.977), respectively. Multimodal models outperform single-modality clinical models in both predictive accuracy and stability.

**Conclusion:**

This study successfully developed a deep learning model based on multiphase enhanced CT images and clinical features to predict risk stratification and prognosis in NB. The findings provide a new tool for clinical practice and lay the foundation for future precision medicine and personalized treatment.

## 1 Introduction

Neuroblastoma (NB) is a common childhood malignancy originating from immature neural crest cells (1). NB is the most common and fatal tumor of infancy, with a median age of diagnosis of 18 months, and accounts for 15% of all childhood cancer-related deaths (2). Despite significant diagnostic and therapeutic advances in recent years, the prognosis of NB remains highly heterogeneous, especially between high-risk (HR) and non-high-risk (NHR) patients (3). Accurate risk stratification is essential for developing personalized treatment plans, as different risk groups require different treatment intensities and strategies (4). Traditional risk stratification methods rely heavily on clinicopathologic features such as age, stage, histologic type, and MYCN amplification status and so on (5). However, these methods are difficult to achieve all of them in the early stage of disease treatment and to meet the clinical needs of early tumor assessment.

In recent years, the application of deep learning in various aspects of oncology research has exploded and made great progress (6). Meanwhile, with the rapid development of medical imaging technology and artificial intelligence, comprehensive tumor assessment strategies based on imaging features have gradually gained attention. As a noninvasive and information-rich imaging technique, multiphase enhanced computed tomography (CT) can provide detailed anatomical and functional information of tumors at different time points, offering new possibilities for risk stratification and prognostic assessment of NB (7). However, traditional imaging analysis methods usually rely on manual interpretation, which is subjective and inconsistent, limiting their application in clinical practice.

To overcome this challenge, this study aims to develop a deep learning-based multi-phase enhanced CT image analysis method for risk stratification and prognostic assessment of NB. We chose Swin Transformer (Swin-T) as the main deep learning architecture because of its excellent performance in tasks such as image classification, target detection, and semantic segmentation (8). Swin-T improves the performance of the model while maintaining high efficiency by restricting the self-attention computation to localized windows through the shift-window strategy, while allowing cross-window connections. In addition, to further improve the accuracy of prognostic assessment, we constructed a comprehensive prognostic model by combining CT image features with clinical features. Through Cox regression analysis and multiple machine learning algorithms, we screened the features related to prognosis and constructed multiple prognostic models. Ultimately, we evaluated the predictive performance of the model by metrics such as C-index, survival analysis, and time-dependent ROC curves, and verified its application value in clinical practice.

This study not only provides a new tool for risk stratification of NB, but also lays the foundation for future precision medicine and individualized treatment. We have developed a promising tool that may provide clinicians with a valuable reference to improve the prognosis and quality of life of NB patients.

## 2 Materials and methods

### 2.1 Case screening

The study was conducted in accordance with the guiding principles of the Declaration of Helsinki, and the study protocol was approved by the Ethics Committee of the First Affiliated Hospital of Zhengzhou University (2024-KY-1031-001). Informed consent was waived due to the retrospective nature of enrollment.

In the electronic medical record database of the First Affiliated Hospital of Zhengzhou University, we comprehensively searched pediatric patients with pathologically confirmed NB admitted from January 2015 to Jul 2024. The case inclusion criteria were as follows: (1) pathologically confirmed NB; (2) preoperative multiphase enhanced CT imaging consisting of at least three phases: non-contrast-enhanced phase (NC phase), arterial phase (ART phase), and portal venous phase (PV phase); (3) available clinical information (including baseline profile, tumor markers, inflammatory markers); and (4) Clear risk stratification information as well as survival status and timing information. Case exclusion criteria were as follows: (1) age older than 14 years; (2) more than 60 days between pathologic examination and preoperative multiphase enhanced CT; and (3) poor image quality. Strictly following the inclusion and exclusion criteria, we collected all cases that met the criteria and randomized them into a training cohort and a testing cohort in a ratio of 8:2.

### 2.2 CT image collection

All included patients underwent 16- or 64-slice spiral computed tomography. Multiphase-enhanced CT images were exported from the image archiving and communication system (PACS) and stored as BMP files. Subsequently, we manually extracted the three-phase CT image slices with the largest tumor cross-sectional area as the CT feature images of the corresponding patients. The manual extraction process was performed by two experienced paediatric surgeons (with >10 years of clinical experience) through consensus review, based on the following criteria: (1) Anatomical coverage: the slice with the largest tumor area in axial view across all phases; (2) Morphological consistency: exclusion of slices with significant artifacts that could distort measurements. In addition, we read the three-phase CT images separately as grayscale maps, and subsequently re-stacked them vertically as three-channel images and imported them into the deep learning model to obtain the joint features of the multi-phase images. Through transform function, we normalized all CT images and converted them to 224 × 224 pixels to fit the inputs of our model.

### 2.3 Classifier structure

Given the strong performance of the Swin-T in image classification, object detection, and semantic segmentation, we choose Swin-T as the primary architecture for our classification task (8). Specifically, we selected a tiny Swin-T subtype to match our small clinical dataset, the detailed structure of which is shown in Supplementary Table S1. The shifted-window strategy of Swin-T restricts self-attention computations to disjoint local windows while also allowing cross-window connections, enhancing both efficiency and effectiveness. We used the pre-training weights of Swin-T on the ImageNet-1 K dataset as the initial weights of the model and modified the final number of output channels of the model from the default of 1,000 to the number of categories in our classification task. To make the output of the model smoother, we added a hidden layer containing 16 neurons between the original output layer of the model and the fully connected layer. At the end of the model, we use the Softmax activation function to normalize the raw scores from the output layer into values between 0 and 1, which serve as the predicted probability values for each class.

### 2.4 Classifier training

In order to prevent data leakage leading to overestimation of model performance, we use the training cohort for the training process of the model and the testing cohort is only used for the performance evaluation of the model. In the training cohort, we applied data enhancement strategies to improve the generalizable performance of the model. Specifically, the original image was rotated as it went between ±10 degrees, with a panning ratio between ±10% in the horizontal and vertical directions, while the image brightness varied randomly between 70 and 130%, and the contrast and saturation varied randomly between 80 and 120%. In the classification task, we used the cross-entropy loss function to compute the loss and the Adam optimizer for model gradient optimization. The cross-entropy loss function is a widely used loss function in classification problems to measure the difference between the probability distribution predicted by the model and the actual probability distribution. The Adam optimizer implements an adaptive learning rate by calculating and storing first-order moment estimates and second-order moment estimates of the gradient, which indirectly mitigates overfitting (9). A cosine learning rate decay strategy was used to adjust the learning rate in a cyclic asymptotic manner, with the maximum number of iterations for cosine annealing set to 20, the maximum learning rate set to 0.00001, and the minimum learning rate defaulted to 0. We configured the batch size to 8 and the maximum number of training epochs to 100. To avoid overfitting and ensure adequate model training, we adopted an early stopping strategy, i.e., when the loss of the testing cohort after training for 25 epochs did not decrease for 10 consecutive epochs, model training was stopped.

In the current study, image preprocessing and classifier construction and training were implemented based on the PyTorch architecture (Python 3.7, PyTorch 1.1.0), and an RTX3090 with 24 GB of memory was used as the GPU to accelerate the training.

### 2.5 Classifier evaluation

The NC phase, ART phase, PV phase images and the integrated multi-phase images are fed into the Swin-T model respectively, and the final classifiers are obtained after a sufficient training process. We then freeze the model weights and comprehensively evaluate the performance of each classifier in the testing cohort. With the ROCR package and the pROC package in R (version 4.4.3), we calculated the area under the receiver operating characteristic curve (AUC) and plotted ROC curves for each model (10, 11). Subsequently, we plotted the confusion matrices of each classifier to obtain a more complete picture of the model’s performance on the different categories. In addition, we calculated sensitivity, specificity, precision, recall value, accuracy, Kappa value and F1 score separately to comprehensively evaluate the performance of each model. In addition, we constructed four classic convolutional neural network (CNN) models using the same training strategy to serve as benchmark models for comparison. After a comprehensive comparison, we selection the model with the best performance as the best classifier for NB risk stratification.

### 2.6 Clinical data collection

To further assess the overall prognosis of NB patients, we decided to include clinical indicators for each tumor patient to construct a comprehensive model combining CT image features with clinical features. The clinical characteristics of the corresponding patient at the time of initial tumor diagnosis were retrieved from the hospital’s information system, which mainly included the patient’s baseline data and tumor markers from hematology tests as well as inflammatory indicators. For qualified clinical indicators, we dealt with missing values through multiple interpolation using the mice package, specifying the interpolation method as “predictive mean matching” (PMM) with an upper limit of 50 iterations (12). Subsequently, we used the Kolmogorov–Smirnov test (KS test) and overlay density plots to verify whether the distribution of the interpolated data was consistent with the non-missing parts of the original data, thereby avoiding bias introduced by interpolation.

### 2.7 Data consolidation

Considering the significant association between risk stratification and overall prognosis of NB patients, we utilized a transfer learning strategy to extract CT image features to assess overall prognosis. This means that we utilized the output values of the hidden layer (containing 16 neurons) of the best classifier as the image features of the corresponding CT image. Subsequently, we integrated the CT image features with the clinical data and applied standardized preprocessing to the combined dataset. This process primarily involved mean normalization and variance scaling to ensure that all data were adjusted to a consistent scale.

### 2.8 Prognosis model construction

We sequentially incorporated the integrated dataset into univariate Cox regression and multivariate Cox regression to initially screen for prognostically relevant features. In the univariate Cox regression analysis, a *p*-value threshold of 0.05 was used for feature selection. However, for the multivariate Cox regression, we raised the threshold to 0.2 to address the challenge of building an effective prognostic model with a limited number of features. Based on the screened patient characteristics, we implemented 79 different combinations of machine learning algorithms using the Mime1 package to obtain the best prognostic model for NB patients (13). Machine learning algorithms involved in these model combinations include Stepwise Cox Regression (StepCox), Randomized Survival Forests (RSF), LASSO Regression, Gradient Boosting Machines (GBM), CoxBoost, Elastic Networks (Enet), Ridge Regression, Partial Least Squares Regression and Cox Regression (plsRcox), Survival Support Vector Machines (survival-SVM), and Supervised Principal Component Analysis (SuperPC).

### 2.9 Prognosis model evaluation

Based on the model prediction results, we separately calculated the C-indexes of the 79 prognostic models in the training cohort and the testing cohort, and we obtained the best-performing prognostic model based on the average C-indexes of the two cohorts. We then calculated prognosis-related machine learning risk scores (MLRS) for each patient through the optimal model and categorized all patients into high and low MLRS groups based on the median value of the MLRS. Kaplan–Meier (K-M) survival analysis was used to evaluate the difference in overall prognosis between the high and low MLRS groups. Meanwhile, we calculated the AUC values of MLRS at 1, 3, 5, and 7 years and plotted the corresponding time-dependent ROC curves. We calculated the confidence interval of the AUC value using the Clopper-Pearson exact interval method, which is based on the binomial distribution and is suitable for small sample sizes. We also constructed a classic LASSO-Cox model based solely on clinical indicators as a benchmark model to demonstrate the added value of multimodal data. Risk stratification and MYCN amplification status are well-recognized prognostic markers for NB patients (14), so we also explored the association between MLRS and risk stratification as well as MYCN amplification status. In addition, we confirmed the significant correlation between MLRS and some tumor markers through Pearson correlation analysis.

## 3 Results

### 3.1 Dataset composition

**Figure 1:**
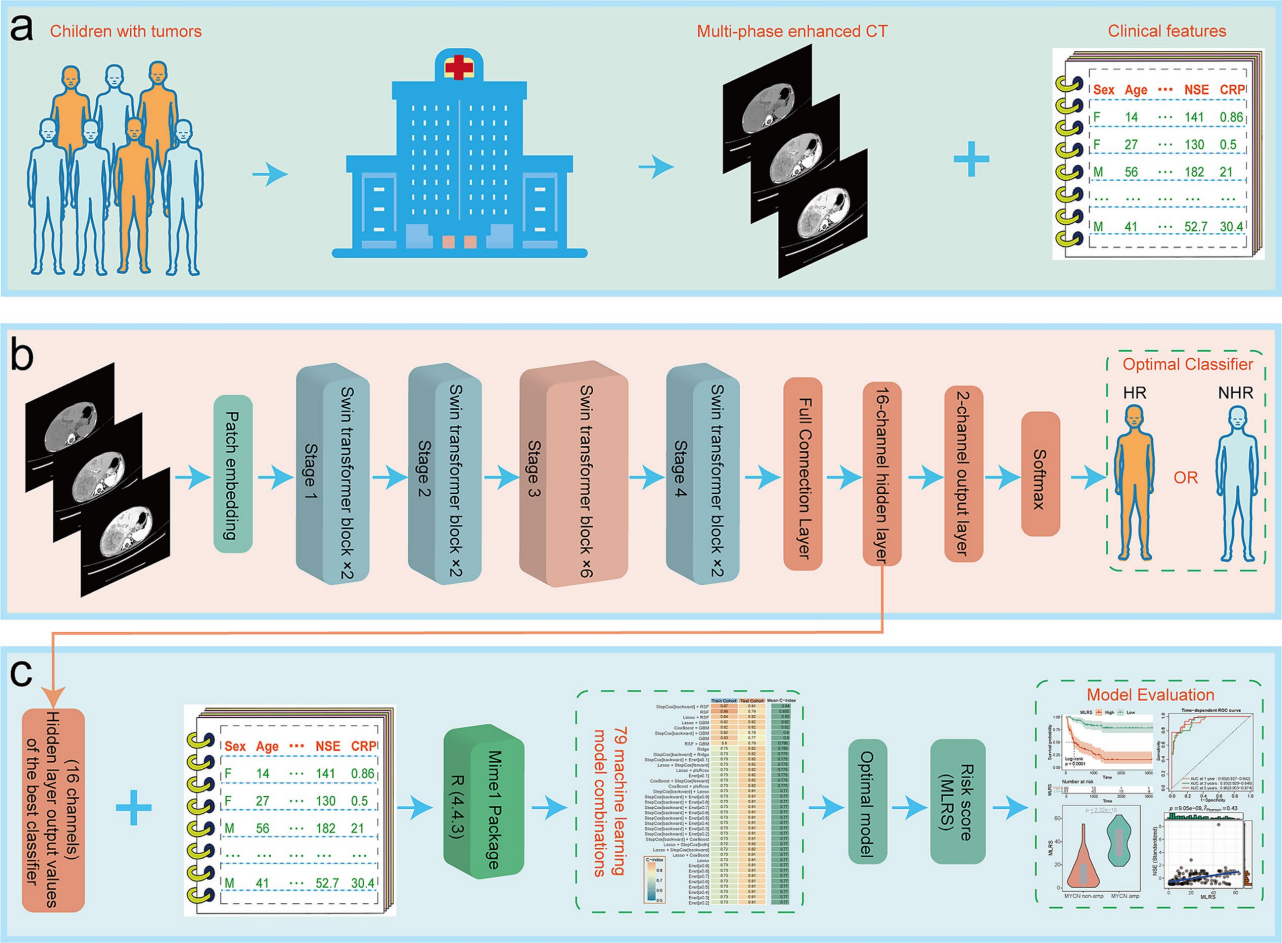
The overall flow of this paper, including **(a)** data collection, **(b)** model structure, and **(c)** some of the important results.

Figure 1 illustrated the overall flow of this paper, including data collection, model structure, and some of the important results. A total of 202 NB patients were enrolled in our study cohort, with 121 HR patients and 81 NHR patients. Eleven indicators have missing values, but the proportion of missing values for all indicators is less than 30% (Supplementary Figure S1a). The D statistics for all variables are well below the empirical threshold of 0.1, indicating that the maximum distance between the cumulative distribution functions of the data before and after interpolation is very small (Supplementary Figure S1b). The overlay density plot also shows that the two curves almost completely overlap, indicating that the distribution of the interpolated values is almost identical to that of the non-missing original values, and that the interpolation process did not introduce any significant bias (Supplementary Figure S1c).

The baseline data for all patients are shown in Supplementary Table S2. Although most baseline characteristics (such as age, gender, survival status, etc.) did not show significant differences between the training and testing cohorts, tumor markers (CA199, CA153) and C-reactive protein (CRP) may have shown random distribution imbalances due to limited sample size (only 41 cases in the testing cohort). The median diagnostic age of all included patients was 38.5 (20.25, 57) months and the median survival time was 657 (232, 1296.75) days. Risk stratification and MYCN amplification status were significantly associated with the overall prognosis of NB patients, whereas there were no significant differences in overall prognosis for different genders or different tumor locations (Supplementary Figure S2).

### 3.2 Optimal classifier for predicting risk stratification

Figure S3 showed typical multi-phase enhanced CT images of NB patients, including the NC phase, ART phase, and PV phase. Each phase of the CT images displayed the characteristics of the tumor at different time points, which were crucial for diagnosis and risk stratification. Additionally, Supplementary Figure S3 illustrated the integrated multi-phase images formed by reading the three-phase CT images as grayscale maps and stacking them vertically. These integrated multi-phase images provided a comprehensive reflection of the tumor changes over different time periods, offering rich information for the deep learning model. By feeding each of the above four types of images into the tailor-made Swin-T model and performing a sufficient training process, we constructed four different risk stratification classifiers: Swin-NC, Swin-ART, Swin-PV, and Swin-MP (representing the multi-phased image model). Supplementary Figure S4 demonstrated the changing process of cross-entropy loss and accuracy of each batch sample during model training as the epoch increased.

**Figure 2:**
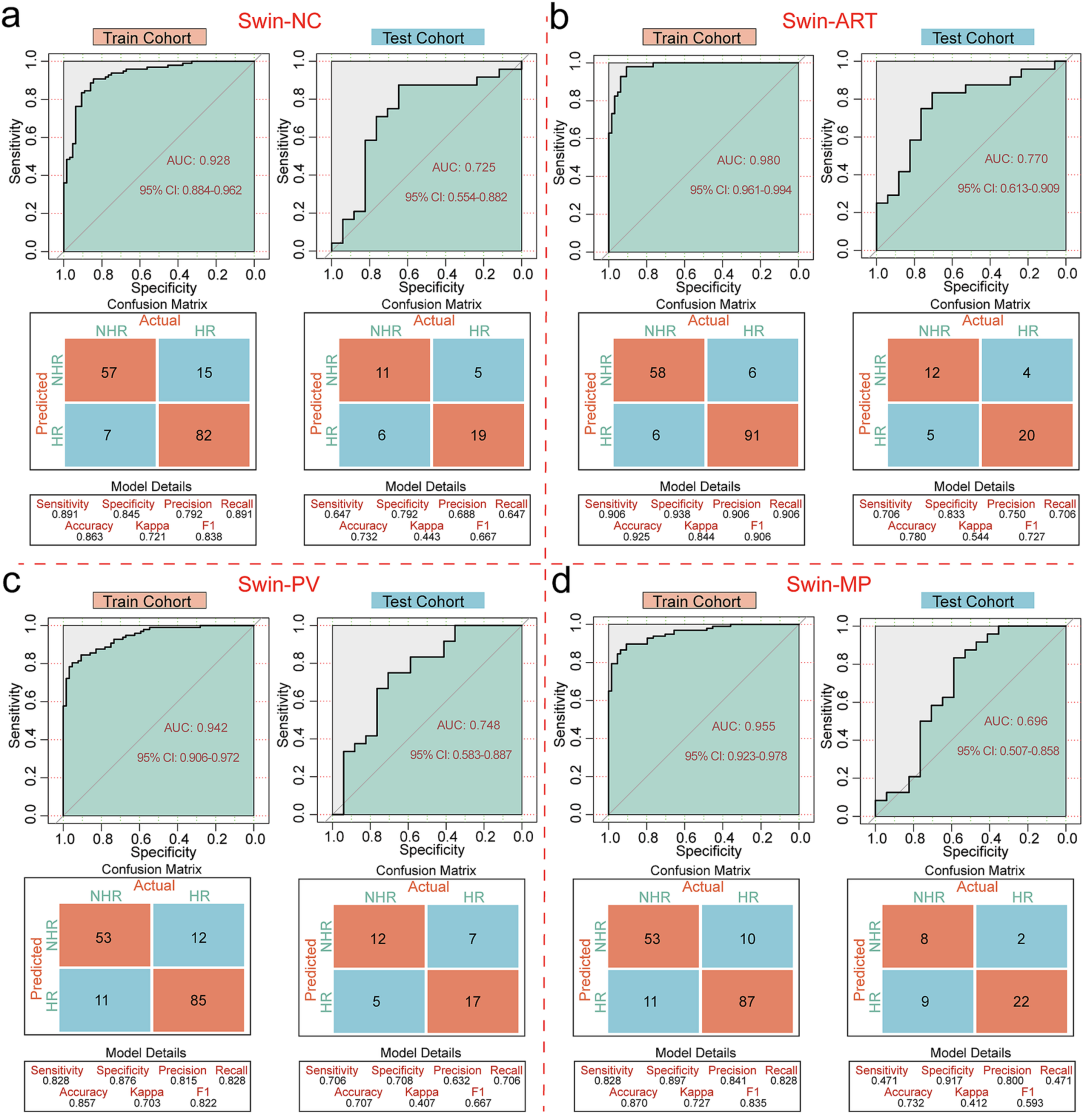
Performance evaluation of risk stratification classifiers. **(a)** ROC curves, confusion matrices and other evaluation metrics for Swin-NC in the training and testing cohorts. **(b)** ROC curves, confusion matrices and other evaluation metrics for Swin-ART in the training and testing cohorts. **(c)** ROC curves, confusion matrices and other evaluation metrics for Swin-PV in the training and testing cohorts. **(d)** ROC curves, confusion matrices and other evaluation metrics for Swin-MP in the training and testing cohorts.

Swin-NC achieved AUC values of 0.928 (95% CI: 0.884–0.962) and 0.725 (95% CI: 0.554–0.882) for the training and testing cohorts, with accuracies of 0.863 and 0.732 (Figure 2a); Swin-ART achieved AUC values of 0.980 (95% CI: 0.961–0.994) and 0.770 (95% CI: 0.613–0.909) for the training and testing cohorts, with accuracies of 0.925 and 0.780 (Figure 2b); Swin-PV achieved AUC values of 0.942 (95% CI: 0.906–0.972) and 0.748 (95% CI: 0.583–0.887) for the training and testing cohorts, with accuracies of 0.857 and 0.707 (Figure 2c); and Swin-MP achieved AUC values of 0.955 (95% CI: 0.923–0.978) and 0.696 (95% CI: 0.507–0.858) for the training and testing cohorts, with accuracies of 0.870 and 0.732 (Figure 2d). In summary, given the strong performance of the Swin-T model in the field of image recognition, all four models achieved excellent prediction performance in the training cohort. Therefore, we mainly compared the performance of the four models in the testing cohort, which reflected the generalization ability of the models. Based on this criterion, Swin-ART possessed the highest AUC value and accuracy in the testing cohort and was considered the best classifier for risk stratification (Figure 2b). In addition, Swin-ART also had a good overall performance, with a sensitivity of 0.706, a specificity of 0.833, a precision of 0.750, a recall value of 0.706, and an F1 score of 0.727 in the testing cohort. Unexpectedly, Swin-MP, which assembled three-phase CT image features, had the worst overall performance, with the lowest AUC value and accuracy in the testing cohort (Figure 2d). We speculated that this might have been due to the fact that integrating three-phase images not only provided more tumor image features but also introduced additional confounding factors that complicated image recognition.

### 3.3 Comparison with CNN classifiers

Based on ART phase CT images, we constructed four classic CNN models: ResNet, DenseNet, AlexNet, and VGGNet, to demonstrate the superiority of the Swin-T model. In the training cohort, the AUC values predicted by the CNN model for risk stratification were all higher than 0.85, but in the testing cohort, they were all lower than Swin-ART, indicating weak generalizability (Supplementary Figure S5).

### 3.4 Optimal model for prognostic assessment

**Figure 3:**
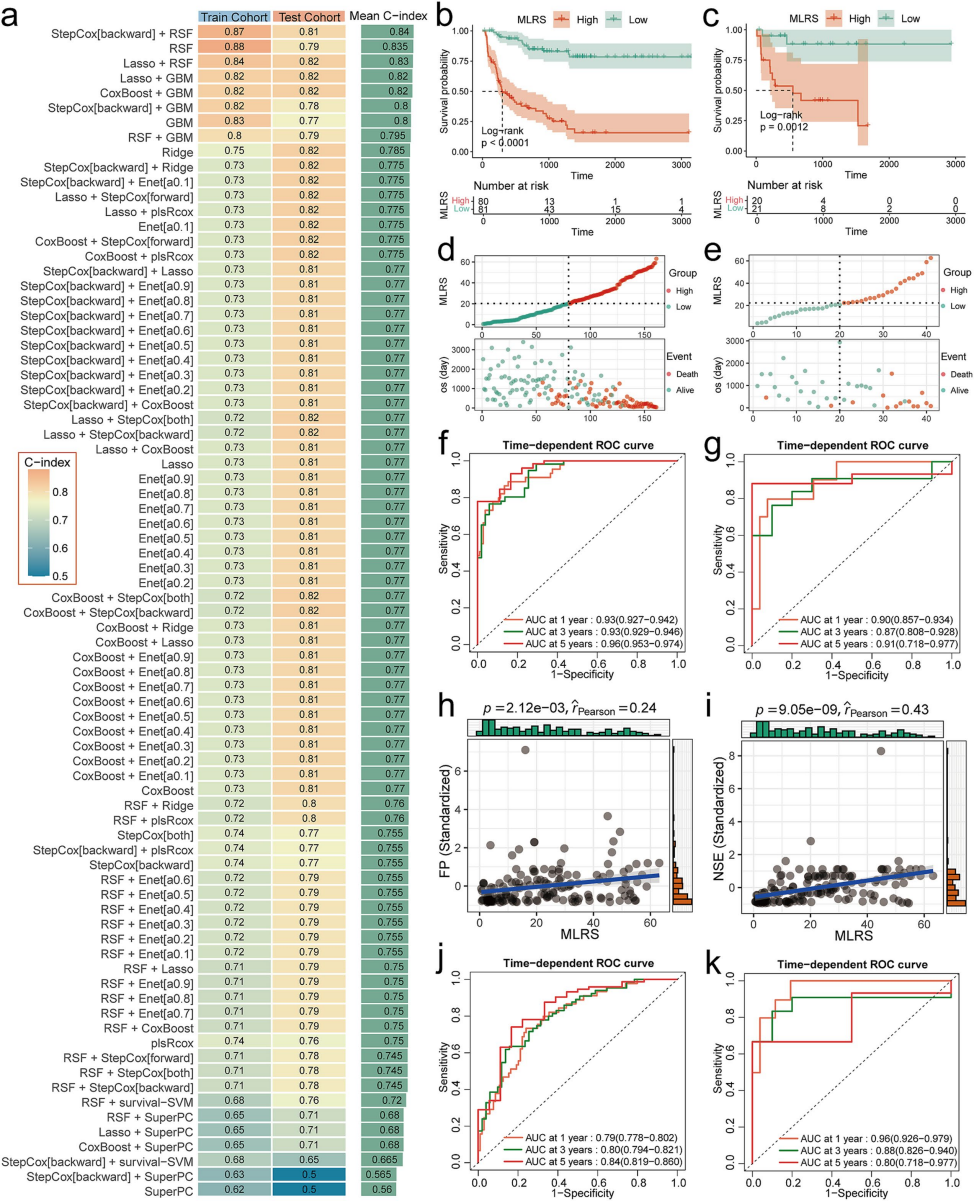
Performance evaluation of prognostic models. **(a)** C-index heatmaps for 79 prognostic models in the training and testing cohorts. **(b,c)** K-M curves for MLRS in training and testing cohorts. **(d,e)** Risk factor correlation plots for MLRS in training and testing cohorts. **(f,g)** Time-dependent ROC curves for MLRS in training and testing cohorts. **(h)** Scatterplot of correlation between MLRS and FP. **(i)** Scatterplot of correlation between MLRS and NSE. **(j,k)** Time-dependent ROC curves for baseline model in training and testing cohorts.

We used the 16-channel data exported from the hidden layer in the Swin-ART model architecture as CT image features and combined them with the clinical characteristics of the patients. Eleven combined features passed the initial screening by Cox regression analysis, containing 6 CT features (hidden layer channels 3, 6, 7, 10, 13, and 15 outputs), MYCN amplification, neuron-specific enolase (NSE), risk stratification, CA153, and CA125 (Supplementary Table S3). In the comparison of prognostic models, the combined model of backward stepwise Cox regression and RSF achieved the highest mean C-index (0. 84), with a C-index of 0.87 in the training cohort and a C-index of 0.81 in the testing cohort, and thus we considered it the best model for prognostic assessment (Figure 3a). Notably, the combined model including RSF occupied the top three places in the ranking of the mean C-index, suggesting the great potential of RSF in assessing the overall prognosis of NB patients.

Subsequently, we calculated the MLRS for each patient based on the best model and comprehensively evaluated its predictive performance. K-M survival analysis showed that patients in the high MLRS group had a significantly (Log-rank *p* < 0.0001 in training cohort and Log-rank *p* = 0.021 in testing cohort) worse overall prognosis than those in the low MLRS group (Figures 3b,c). Patients in the high MLRS group also experienced significantly higher mortality than those in the low MLRS group (Figures 3d,e). The 1-, 3-, and 5-year AUC values of the MLRS for predicting overall survival in the training cohort were 0.93 (95% CI: 0.927–0.942), 0.93 (95% CI: 0.929–0.946), and 0.96 (95% CI: 0.953–0.974), respectively (Figure 3f). The corresponding AUC values for the testing cohort were 0.90 (95% CI: 0.857–0.934), 0.87 (95% CI: 0.808–0.928), and 0.91 (95% CI: 0.718–0.977), respectively (Figure 3g). Decision curve analysis (DCA) also demonstrated that MLRS can achieve high net clinical benefit for NB patients (Supplementary Figure S6). Additionally, MLRS was strongly associated with existing prognostic markers. The Wilcoxon test showed that the overall distribution of MLRS was significantly (*p* < 1.6e-15) higher in HR patients compared to NHR patients, and significantly (*p* < 2.22e-16) higher in MYCN-amplified patients compared to MYCN non-amplified patients (Supplementary Figure S7). In correlation analysis, MLRS showed significant positive associations with both Ferritin (FP) and NSE (Figures 3h,i).

## 4 Performance gains from multimodality

The benchmark model achieved C-index values of 0.74 and 0.84 in the training and testing cohorts, respectively. The abnormal jump in predictive performance from the training to the testing cohort may be due to biased distribution of clinical variables and small sample size in the testing cohort. The 1-, 3-, and 5-year AUC values of the benchmark model in the training cohort were 0.79 (95% CI: 0.778–0.802), 0.80 (95% CI: 0.794–0.821), and 0.84 (95% CI: 0.816–0.860), respectively (Figure 3j). The corresponding AUC values for the testing cohort were 0.96 (95% CI: 0.926–0.979), 0.88 (95% CI: 0.826–0.940), and 0.80 (95% CI: 0.718–0.977), respectively (Figure 3k). Compared with the baseline model, MLRS performs more stably between the training and testing cohorts, and the small difference between the training and testing sets indicates a low risk of overfitting. In the testing cohort, the predictive performance of MLRS also showed longitudinal temporal stability, while the performance of the benchmark model declined significantly over time, suggesting that MLRS is suitable for long-term prognosis prediction (Figures 3g,k).

## 5 Discussion

In this study, we successfully predicted the risk stratification and overall prognosis of NB patients by developing a deep learning model based on multiphase enhanced CT images and clinical features. The results showed that Swin-ART performed well in risk stratification prediction with an AUC value of 0.770 and an accuracy of 0.780 on the testing cohort. In addition, the integrated prognostic model combining CT image features and clinical features achieved a C-index of 0.81 on the testing cohort, which demonstrated a good prediction performance. These findings not only provide a new tool for risk stratification of NB, but also lay the foundation for personalized treatment and prognostic assessment in clinical practice.

The Swin-ART model performed best on multiphase enhanced CT images, especially outperforming the other models on the testing cohort. This result may be attributed to the importance of ART phase images in early tumor angiogenesis, which can more accurately reflect tumor biology. A radiomics study based on ART phase images by Wang et al. identified subgroups of ultra-high-risk patients from HR patients to help predict early disease progression (15). These findings demonstrated the significant value of ART images in the early evaluation of NB patients. In contrast, the Swin-MP model did not perform as well as expected despite integrating image information from three periods. This may be due to the fact that multi-phase images introduce more noise and interfering factors, leading to a decrease in the generalization ability of the model. Therefore, in practical applications, choosing appropriate image phases is crucial to improve the model performance.

The comprehensive prognostic model combining CT image features and clinical features performed well on several assessment metrics. In particular, the C-index of the model based on backward stepwise Cox regression and RSF reached 0.81 on the test cohort, indicating that the model had high predictive accuracy. In addition, K-M survival analysis showed that patients in the high MLRS group had a significantly worse prognosis, further validating the validity of the model. Interestingly, MLRS was significantly positively correlated with both FP and NSE, and both demonstrated an elevated trend in the group of patients with poorer prognosis. Iron is an essential metal for cellular metabolism and maintains iron homeostatic regulation under normal body conditions. Cancer cells exhibit iron homeostatic dysregulation and, for underlying reasons, require more iron for metabolism and growth (16). And NSE is an important tumor marker in NB patients, and serum NSE levels greater than 100 ng/mL are associated with poor prognosis (17). MLRS outperforms single-modality clinical models in both predictive power and stability, demonstrating the value-added effects of multimodal models. These results demonstrate the importance of multimodal data fusion in prognostic assessment to provide more comprehensive and accurate predictions.

Multimodal data fusion in tumor patients is one of the focuses of future early tumor assessment. In addition to CT images and clinical features, the fusion of other imaging technologies (e.g., MRI, PET-CT) and biomarkers (e.g., gene expression profiles, proteomics data) can be explored in the future to further improve the predictive performance of the model. The integrated analysis of multimodal data will provide more comprehensive information for risk stratification and prognostic assessment of NB. Future studies could also explore dynamic monitoring models based on time-series data to assess patients’ condition changes and treatment effects in real time. By combining patients’ clinical data and imaging characteristics, personalized treatment recommendations can be provided for each patient to further improve treatment outcomes and survival rates.

Despite the remarkable results of this study, there are still some limitations. First, the study sample size is relatively small, and larger multicenter studies are needed in the future to verify the stability and generalization ability of the model. Second, only CT images were used in this study, and other imaging techniques can be explored for NB risk stratification and prognostic assessment in the future. In addition, the interpretability of the model still needs to be improved, and the decision-making mechanism of the model can be further revealed in the future through visualization techniques and feature significance analysis to enhance clinicians’ trust.

## 6 Conclusion

In summary, this study successfully achieved risk stratification and prognostic assessment of NB by developing a deep learning model based on multiphase enhanced CT images and clinical features. The findings not only provide new tools for clinical practice, but also lay the foundation for future precision medicine and individualized treatment. Future studies will further expand the sample size and explore more imaging techniques and model interpretability to improve the utility and clinical value of the model.

## Data Availability

The original contributions presented in the study are included in the article/Supplementary material, further inquiries can be directed to the corresponding author/s.
