# Supplementary figures and images for: Development of intelligent tools to predict neuroblastoma risk stratification and overall prognosis based on multiphase enhanced CT and clinical features
Source: Front Neurol. 2025 Jun 19;16:1573398. doi: 10.3389/fneur.2025.1573398 (PMC12221888; doi:10.3389/fneur.2025.1573398)

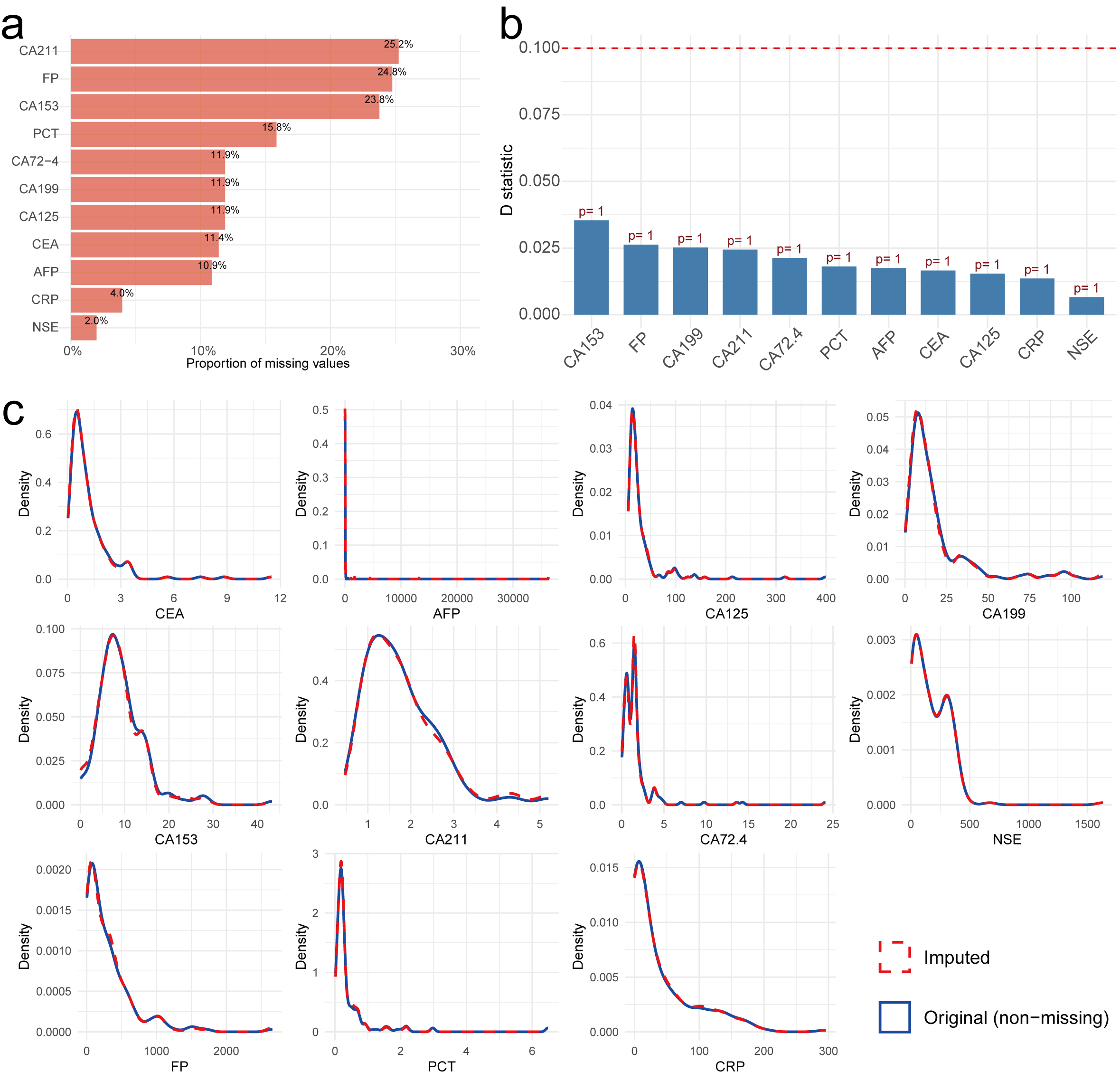

Supplement: Supplementary file 1 [file Data_Sheet_1.ZIP › Figure S1.tif]

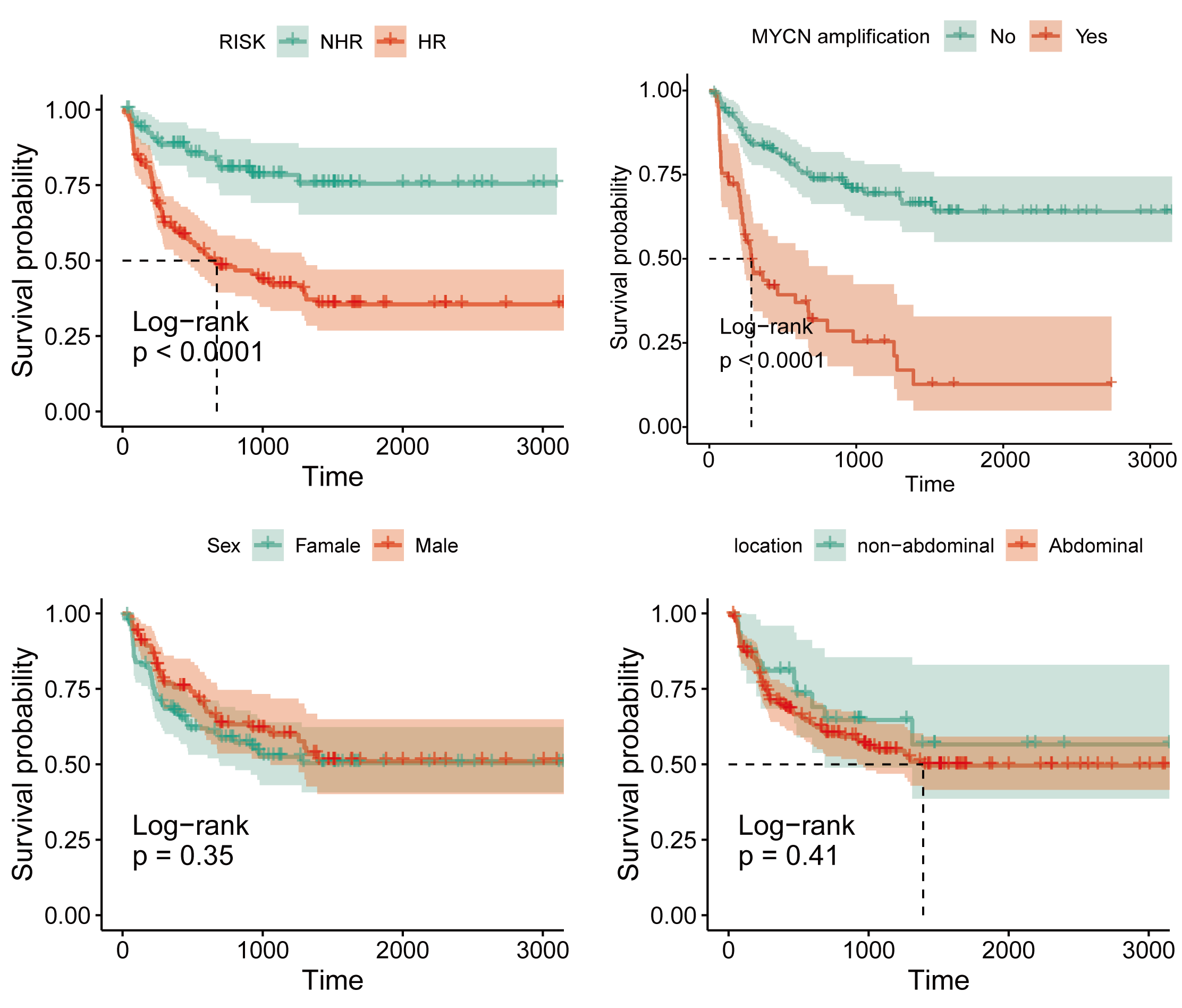

Supplement: Supplementary file 1 [file Data_Sheet_1.ZIP › Figure S2.tif]

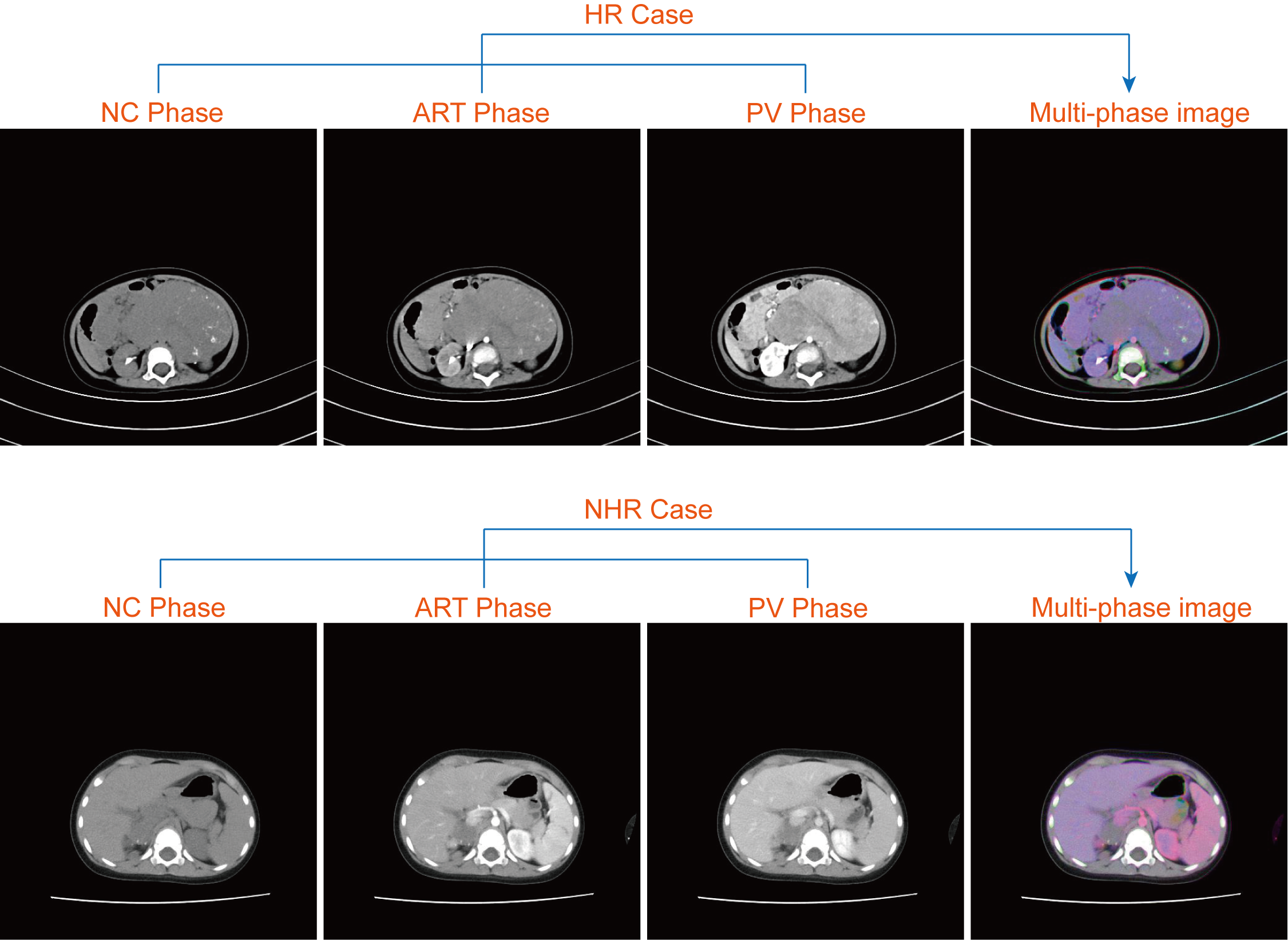

Supplement: Supplementary file 1 [file Data_Sheet_1.ZIP › Figure S3.tif]

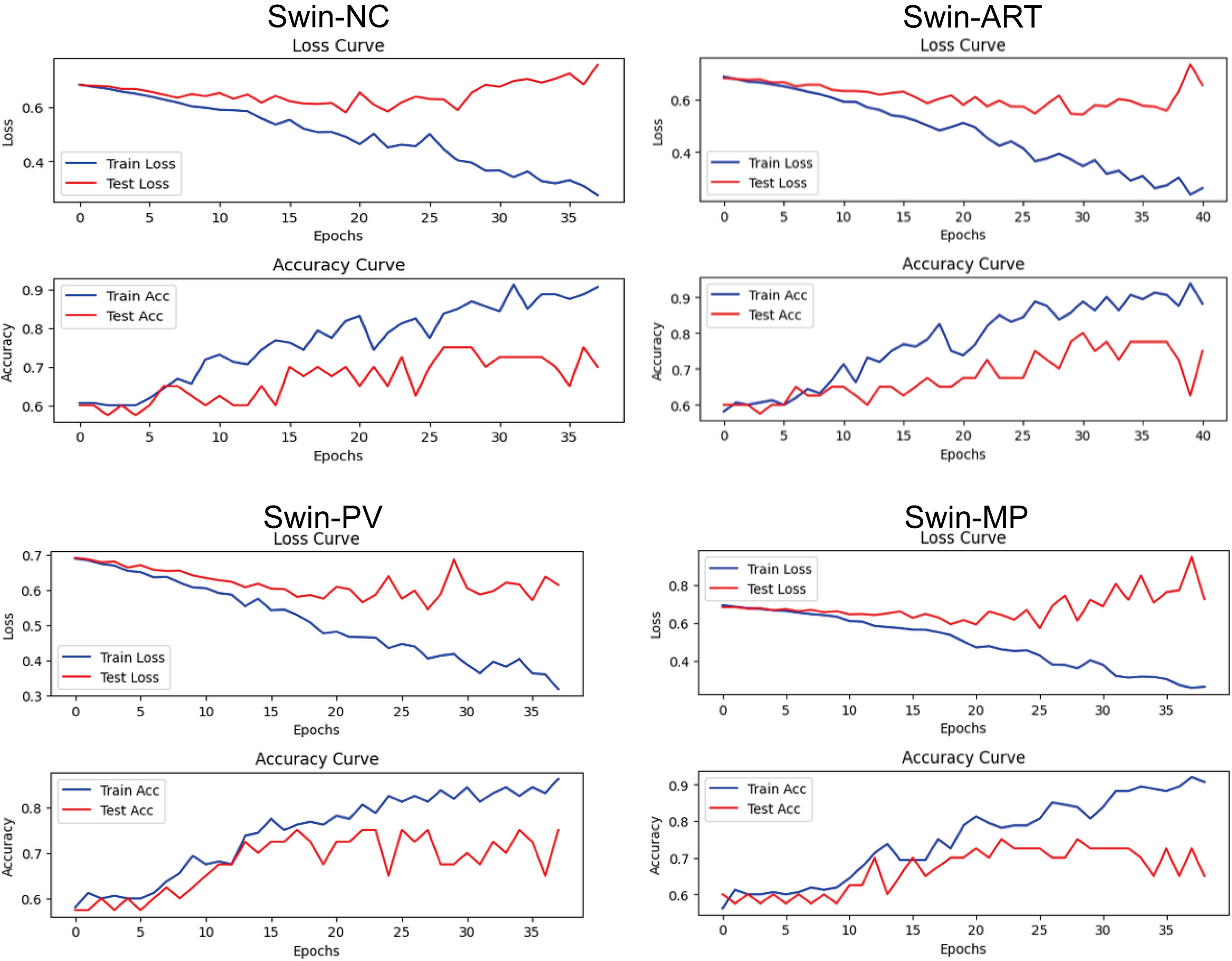

Supplement: Supplementary file 1 [file Data_Sheet_1.ZIP › Figure S4.tif]

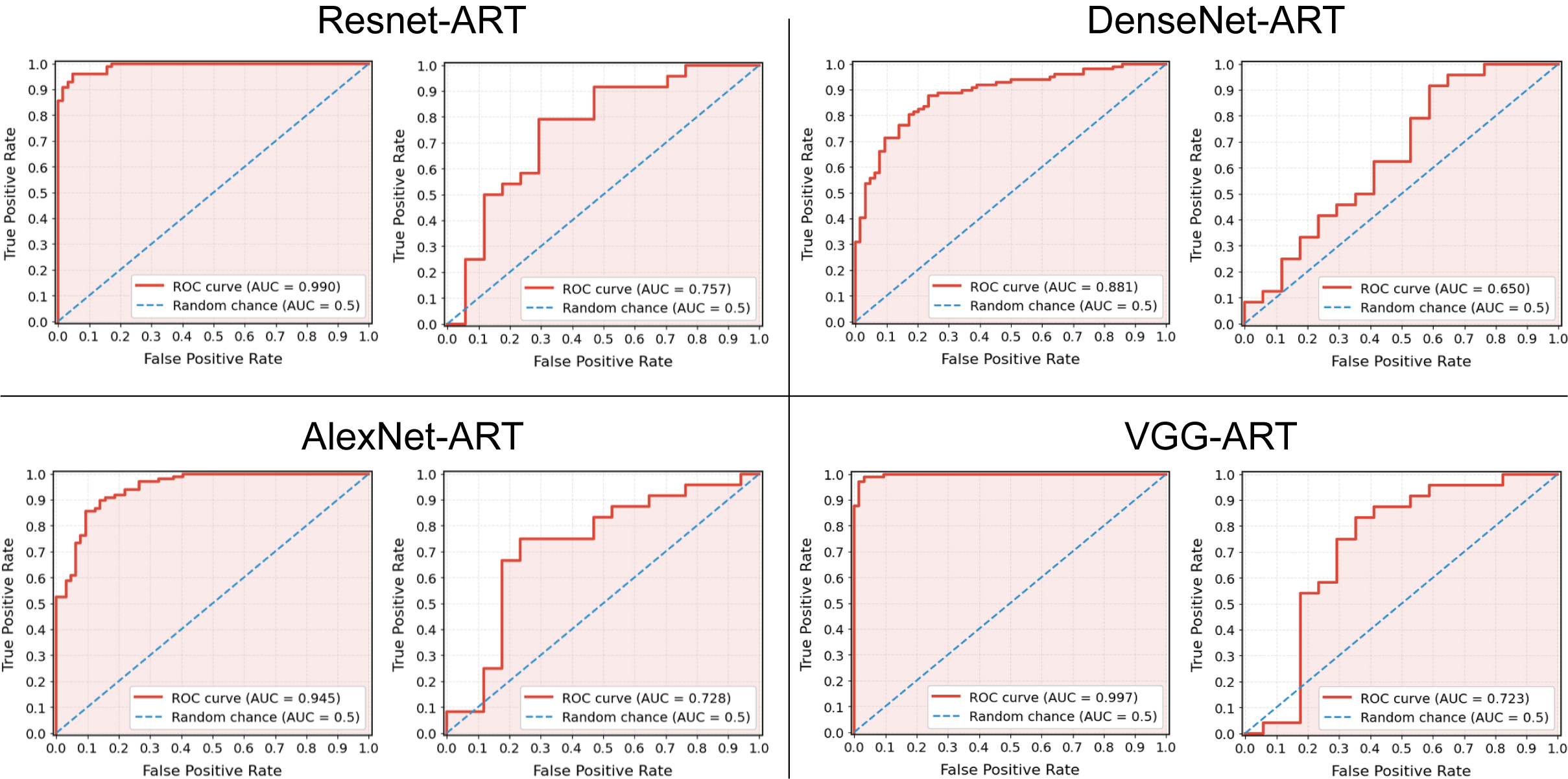

Supplement: Supplementary file 1 [file Data_Sheet_1.ZIP › Figure S5.tif]

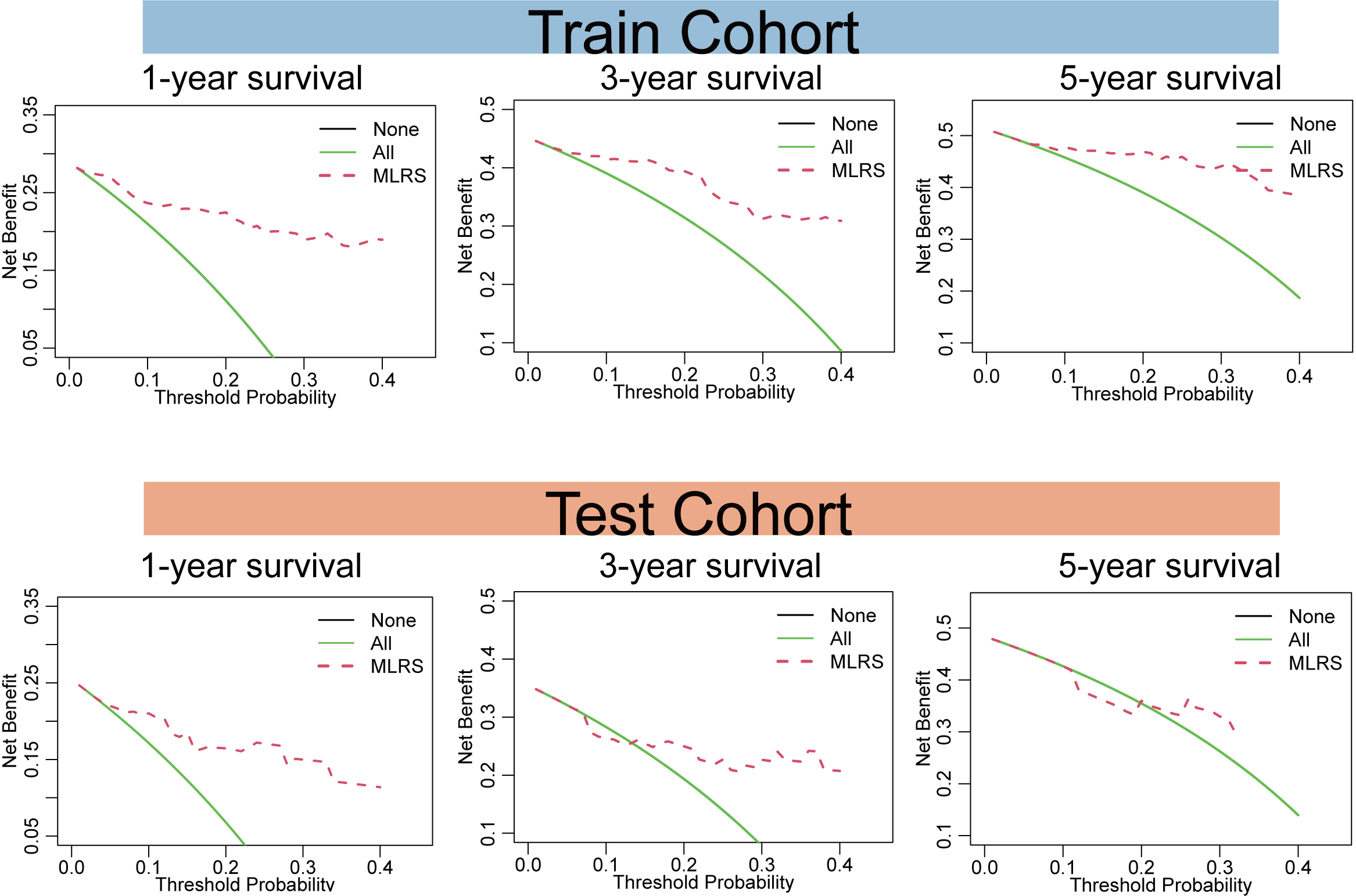

Supplement: Supplementary file 1 [file Data_Sheet_1.ZIP › Figure S6.tif]

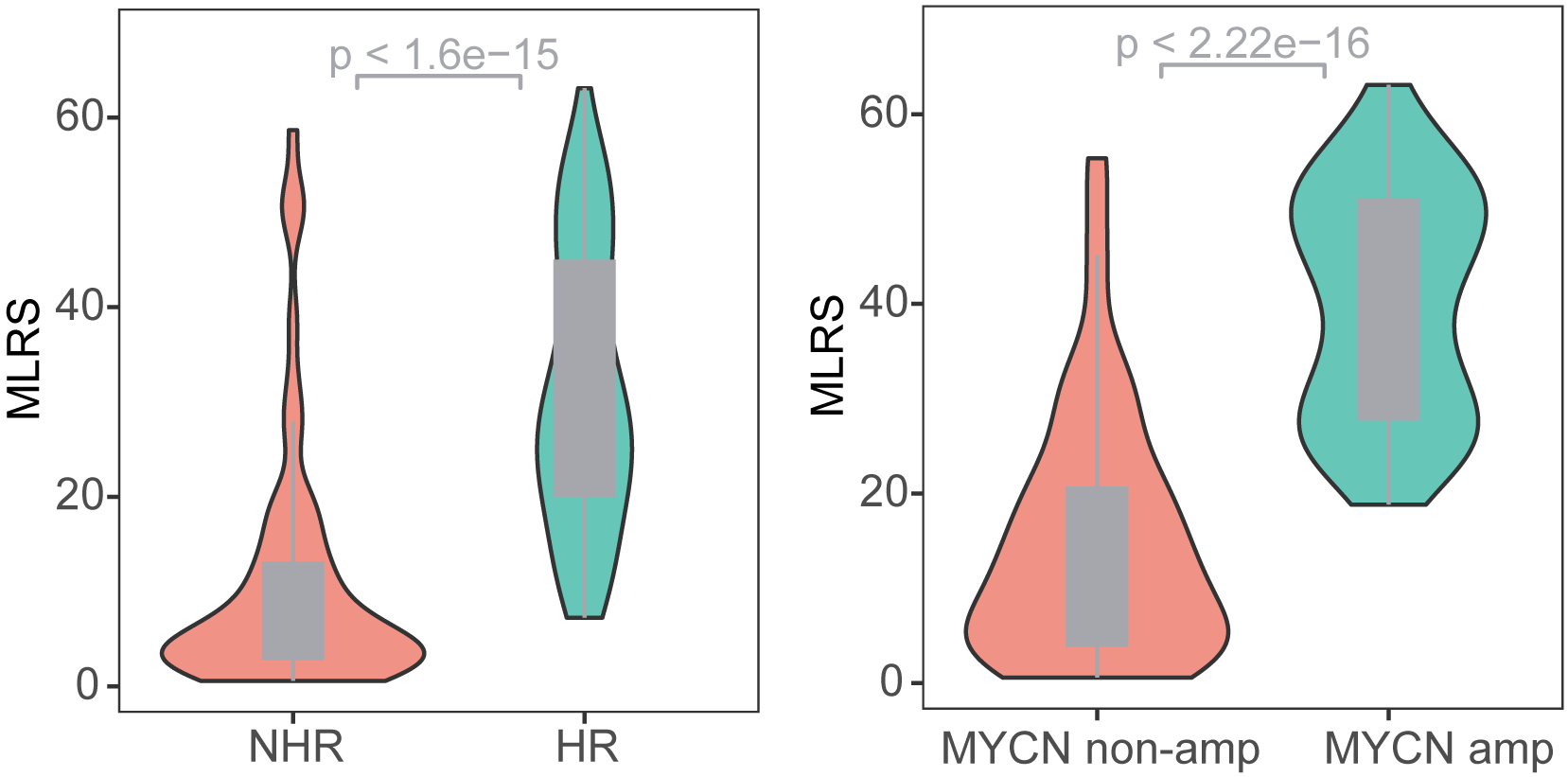

Supplement: Supplementary file 1 [file Data_Sheet_1.ZIP › Figure S7.tif]
